# CD4+ROR***γ***t++ and Tregs in a Mouse Model of Diet-Induced Nonalcoholic Steatohepatitis

**DOI:** 10.1155/2015/239623

**Published:** 2015-07-01

**Authors:** Luisa Vonghia, Nathalie Ruyssers, Dorien Schrijvers, Paul Pelckmans, Peter Michielsen, Luc De Clerck, Albert Ramon, Emilio Jirillo, Didier Ebo, Benedicte De Winter, Chris Bridts, Sven Francque

**Affiliations:** ^1^Department of Gastroenterology and Hepatology, Antwerp University Hospital, 2560 Antwerp, Belgium; ^2^Department of Basic Medical Sciences, Neuroscience and Sensory Organs, University of Bari, 70100 Bari, Italy; ^3^Laboratory of Experimental Medicine and Paediatrics, Division of Gastroenterology, University of Antwerp, 2560 Antwerp, Belgium; ^4^Laboratory of Pharmacology, University of Antwerp, 2560 Antwerp, Belgium; ^5^Laboratory of Immunology, Allergology and Rheumatology, University of Antwerp, 2560 Antwerp, Belgium; ^6^International Tissue Engineering Research Association (ITERA) Life Sciences Forum, 3620 Lanaken, Belgium; ^7^Genetisch-Diagnostisches Labor, 50939 Köln, Germany

## Abstract

*Background and Aims*. Inflammatory mediators that cross-talk in different metabolically active organs are thought to play a crucial role in the pathogenesis of Nonalcoholic Steatohepatitis (NASH). This study was aimed at investigating the CD4+ROR*γ*t+ T-helper cells and their counterpart, the CD4+CD25+FOXP3+ regulatory T cells in the liver, subcutaneous adipose tissue (SAT), and abdominal adipose tissue (AAT) in a high fat diet (HFD) mouse model. *Methods*. C57BL6 mice were fed a HFD or a normal diet (ND). Liver enzymes, metabolic parameters, and liver histology were assessed. The expression of CD4+ROR*γ*t+ cells and regulatory T cells in different organs (blood, liver, AAT, and SAT) were analyzed by flow cytometry. Cytokine and adipokine tissue expression were studied by RT-PCR. *Results*. Mice fed a HFD developed NASH and metabolic alterations compared to normal diet. CD4+ROR*γ*t++ cells were significantly increased in the liver and the AAT while an increase of regulatory T cells was observed in the SAT of mice fed HFD compared to ND. Inflammatory cytokines were also upregulated. *Conclusions*. CD4+ROR*γ*t++ cells and regulatory T cells are altered in NASH with a site-specific pattern and correlate with the severity of the disease. These site-specific differences are associated with increased cytokine expression.

## 1. Introduction

Many efforts have been undertaken to unravel the role of the immune system in the pathogenesis of Nonalcoholic Steatohepatitis (NASH) [1]. The balance between proinflammatory Th17 and the regulatory T cells (Tregs), which plays a major role in the control of inflammation, appears to be disturbed in various diseases, such as autoimmune, infectious, inflammatory, and metabolic diseases [2]. The role of these cells in NASH has not yet been fully clarified.

Th17 are a subset of effector T cells that express the nuclear receptor retinoid-related orphan receptor *γ*t (ROR*γ*t) and produce IL17. An upregulation of the Th17 pathway has been described in the liver in fatty liver disease [3] and in liver fibrosis, which is the hallmark of progressive liver disease [4]. Opposite to these findings, a protective role of IL17 was described in obesity, since IL17 acted as a negative regulator of adipogenesis and glucose metabolism in mice and delayed the development of obesity [5]. Accordingly, a reduction of Th17 in abdominal adipose tissue (AAT) was described in mice fed a high fat diet (HFD) [6].

Tregs derive from CD4+ Th0 cells and constitutively express CD25 (the IL2 receptor *α* chain). In addition, they express FOXP3 (forkhead/winged helix transcription factor) that is crucial for their function [7]. In a HFD mouse model, a liver-specific and reversible depletion of Tregs was observed [8], while human studies showed their increase in liver biopsies of NASH patients with a more severe disease [9].

Moreover, Tregs were specifically reduced in the AAT of preclinical insulin-resistant models of obesity [6, 10], while in obese patients some authors described an increase of FOXP3 RNA in the subcutaneous adipose tissue (SAT) [10] and others found a downregulation of FOXP3 RNA only in obese patients without insulin resistance, while no difference was found when comparing insulin-resistant obese patients and lean controls [11].

Other key factors in the pathogenesis of NASH are the adipokines, which are mainly produced in the white adipose tissue. Leptin is able to decrease appetite and concomitantly increases energy expenditure [12]. In obese patients, leptin resistance leads to a compensatory increase in leptin levels. High to normal serum leptin levels can be found in NASH patients independently from the body mass index (BMI) [13]. Leptin has also important proinflammatory effects, acting both on the innate and on the adaptive immunity [14, 15]. Recent lines of evidence have shown that leptin is implicated in the upregulation of the Th17 pathway [16, 17] and in the suppression of Treg proliferation [18].

In order to further elucidate the differential role of Th17 and Tregs at different anatomical sites in the pathogenesis of NASH, we analyze in the present study the Treg and Th17 populations in mice fed HFD and their location-specific modifications with particular interest in liver and AAT and SAT. Moreover we analyze the possible correlation between the tissue-specific variations of these cells and the diet-induced disturbances of metabolic homeostasis.

## 2. Materials and Methods

### 2.1. Mice

C57BL6/J six-to-eight-week-old male mice were purchased (Charles River Laboratories, Brussels, Belgium) and kept at the animal facility of the University of Antwerp in temperature- and light-controlled conditions. Mice were allowed to consume water and pellet chow ad libitum.

Mice were fed for 36 weeks with either HFD (with 60% of kcals derived from fat, 20% from proteins, and 20% from carbohydrate) (Research Diets) or normal chow, in line with what was previously described [10]. Mice were weighed weekly and just prior to sacrifice. At sacrifice blood samples, SAT, epididymal fat tissue and liver were obtained. In mice epididymal fat is representative of AAT [10]. Epididymal fat is able to influence the major components of the metabolic syndrome, as shown by the improvement of insulin sensitivity and the decrease of leptin secretion by SAT after its removal [19].

### 2.2. Ethics Statement

The protocols were approved by the Antwerp University Ethical Committee on Animal Experiments (permit number: 2012-47). The animals received human care and were treated according to the Helsinki declaration, the national guidelines for animal protection, and the “Guide for the Care and Use of Laboratory Animals” (National Institutes of Health, 1985).

### 2.3. Cell Preparation and Flow Cytometry

Mice were anesthetized with pentobarbital. Blood samples on ethylenediaminetetraacetic acid (EDTA) were collected from anesthetized mice by cardiac puncture. SAT and AAT and liver were excised after flushing organs through the right ventricle with 5 mL of cold phosphate buffered saline (PBS) (Sigma) and cut into small pieces of 1-2 mm. Liver tissue was smashed through a sieve and intrahepatic immune cells were harvested after centrifugation with 33% Percoll (Sigma) and heparin [20]. Fat tissue fragments were incubated for 60 minutes with collagenase type II (Sigma), filtered, and incubated with DNAse I (Sigma) for 20 min [21].

The cells were incubated with the following fluorochrome conjugated antibodies: CD45-eF450 (BD), CD3-PerCPCy5.5 (Bio-Legend), CD4-FITC (e-Bioscience), and CD25-AlexaFluor700 (e-Bioscience). The cells were fixed in Phosflow, Lyse Fix (Becton Dickinson). Intracellular stains for FOXP3-APC (e-Bioscience) and ROR*γ*t-PE (e-Bioscience) were performed using the FOXP3 Staining Buffer Set (e-Bioscience) according to the manufacturer's instructions. All antibodies were titered before use.

Samples were measured on a FASCanto II flow cytometer with DIVA software (BD) and analyzed with Kaluza software (Beckman Coulter). Lymphocytes were selected based on side scatter and CD45 positivity. T lymphocytes were selected as CD45+CD3+. Among this population, Tregs were defined as CD4+CD25+FOXP3+ and Th17 cells as CD4+ROR*γ*t++. The different populations were defined as the level of the positive population above the 99th percentile of a fluorescence-minus-one (FMO) sample. T-cell populations were expressed in percent of the gated population [22].

### 2.4. Histology

After fixation and sectioning, the liver tissue samples were stained with Haematoxylin-Eosin and Trichrome-Masson. Steatosis, lobular inflammation, and ballooning were scored in a blinded way by one single experienced hepatologist according to the NASH Clinical Research Network Scoring System. The fibrosis grade was also assessed [23]. The NASH Activity Score (NAS) was calculated as the sum of the scores for steatosis, ballooning, and lobular inflammation [24]. NASH was diagnosed if some degree of steatosis, lobular inflammation, and ballooning were simultaneously present [24, 25].

### 2.5. Isolation of mRNA

Total RNA was extracted from stored frozen liver samples using the column-based technique (RNeasy Minikit, Qiagen, KJ Venlo, Netherlands), according to the manufacturer's instructions. The total RNA extraction for the SAT and AAT samples was based on the Trizol (Invitrogen, Life Technology, Belgium) procedure from W. M. Keck Foundation Biotechnology Microarray Resource Laboratory at Yale University. Purified total RNAs were treated with DNase to obtain DNA-free RNA (Turbo DNase free, Ambion, Life Technology, Belgium). cDNA was synthesized using a Transcriptor First Strand cDNA Synthesis Kit (Roche Applied Science, Indianapolis, IN).

### 2.6. Reverse Transcriptase PCR (RT-PCR)

TaqMan Gene expression assay for IL10 (Life Technologies, Belgium, Gene ID 16153, reference Mm00439614_m1), IL17A (Life Technologies, Belgium, Gene ID 16171, reference Mm00439618_m1), and leptin (Gene ID 16846, reference Mm00434759_m1) was performed on an ABI Prism 7300 sequence detector system (Applied Biosystems, Life Technology, Belgium) in 25 *μ*L reaction volumes containing 2 *μ*L cDNA and 23 *μ*L master mix. The master mix was prepared using 12.5 *μ*L TaqMan Universal PCR master mix (Life Technologies, Belgium), 1.25 *μ*L primer, and 9.25 *μ*L RNaseDnase free water (Gibco, Life Technologies, Belgium). Preincubation was performed at 50°C during 2 minutes and 95°C during 10 min. The parameter for PCR amplification was followed by 50 cycles of 95°C for 15 s (denaturation) and 60°C for 1 minute (annealing and extension). Glyceraldehyde 3-phosphate dehydrogenase (GADPH) was used as a housekeeping gene to normalize the results (Life Technologies, Belgium, Gene ID 14433, reference Mm99999915_g1). The relative mRNA expression of each studied gene was calculated using the ΔCt method [26]. The Ct value corresponds to the number of cycles to reach a defined threshold and therefore it increases with a decreasing amount of the template. The ΔCt is the Ct value for any sample normalized to the housekeeping gene.

### 2.7. Serum Tests

Alanine aminotransferase (ALT) was measured by means of enzyme-linked immunosorbent assay (ELISA). All samples were analyzed on the same day. Fasting glucose was measured with a blood glucose-meter (Accu Check).

### 2.8. Statistics

The data are presented as median (range). The Mann-Whitney *U* test and Student's *t*-test were used to compare independent variables, as appropriate. Correlations were calculated with the Spearman's rank correlation coefficient. SPPS 20.0 was used for all statistical calculations.

## 3. Results

### 3.1. Validation of the Model

**Figure 1:**
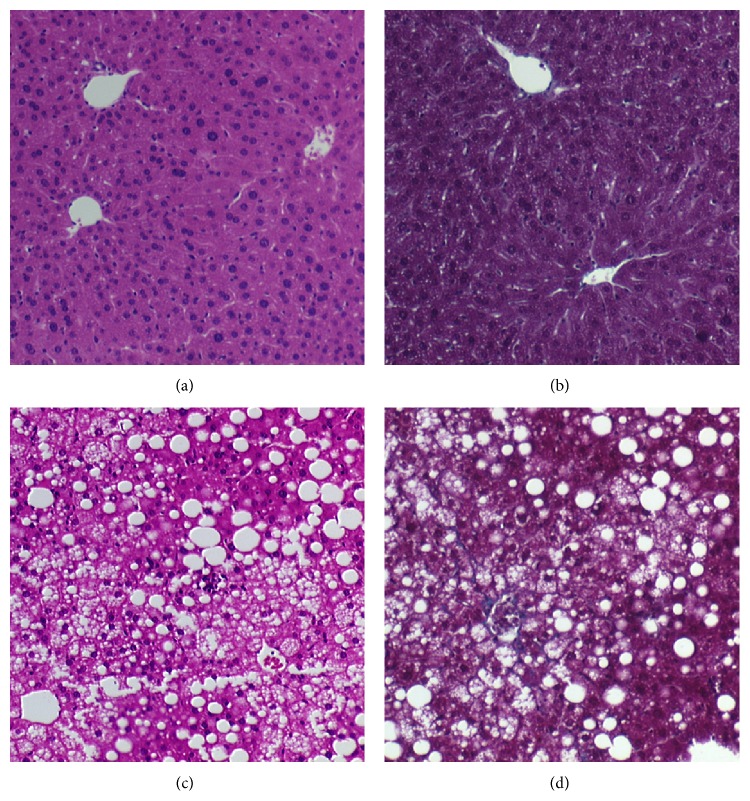
Liver histology in mice fed normal diet (ND) ((a) Hematoxylin/Eosin stain; (b) Masson's Trichrome stain, original magnification ×10) or high fat diet (HFD) ((c) Hematoxylin/Eosin stain; (d) Masson's Trichrome stain, original magnification ×10). Mice fed ND showed a normal liver histology while mice fed HFD showed steatosis, lobular inflammation, and ballooning and therefore proved the presence of Nonalcoholic Steatohepatitis (NASH).

Table 1 summarizes the main characteristics to validate the model. Weight gain in mice fed HFD was 5-fold more than mice fed ND. Liver histology showed steatosis, lobular inflammation, and ballooning in the mice fed HFD and therefore confirmed the presence of NASH. The mice fed a ND showed normal histology (Figure 1). Moreover the blood tests showed higher fasting glucose levels and ALT levels in the HFD group in comparison with the ND group.

These data confirmed the validity of the experimental model to obtain HFD-induced NASH together with an impairment of glucose metabolism and obesity-like disturbances.

### 3.2. Flow Cytometry

Flow cytometry analysis is summarized in Table 2. No modifications in the percentage of leukocytes (CD45+ cells), T-lymphocytes (CD45+CD3+ cells), and CD4+ T-lymphocytes were found in mice fed a HFD in comparison with the lean counterparts, except for a decrease of the CD4+ in the AAT after HFD.

**Figure 2:**
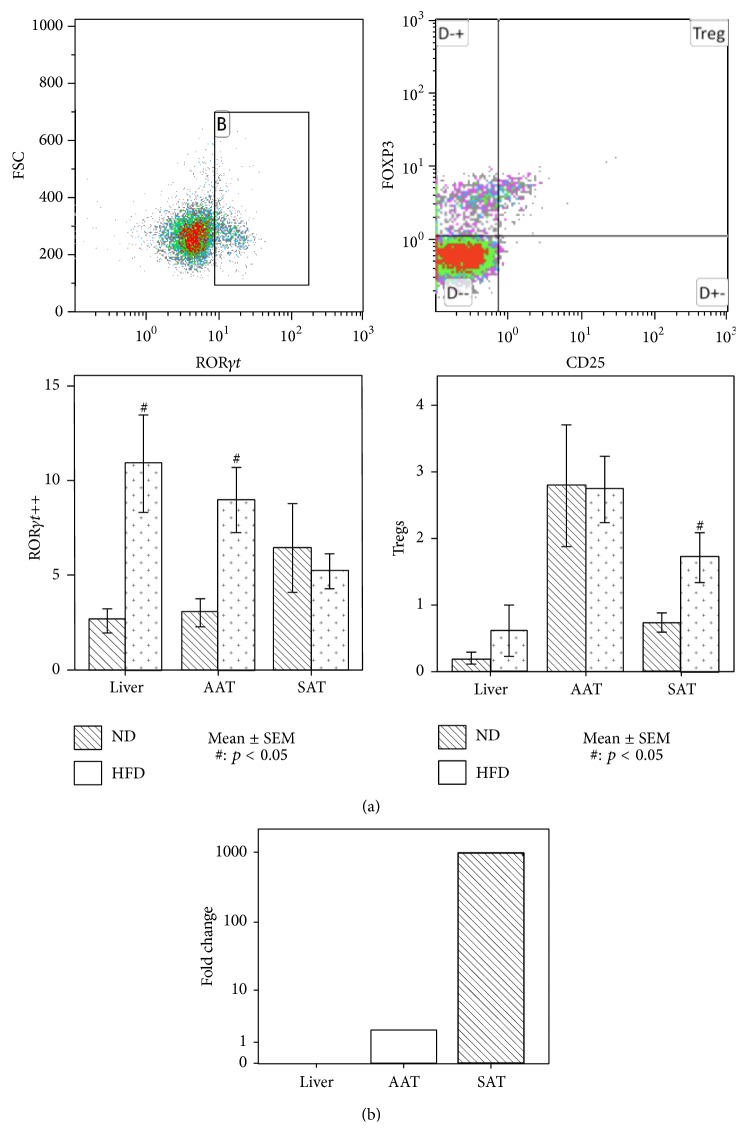
(a) Representative flow cytometry plots of CD4+ROR*γ*t++ and Tregs. CD4+ROR*γ*t++ were selected as the population with bright positivity for ROR*γ*t within the CD45+CD3+CD4+ cells. Tregs were selected as the CD25+FOXP3+ population within the CD45+CD3+CD4+ cells. The bar plots represent the frequency of CD4+ROR*γ*t++ and Tregs in liver, abdominal fat, and subcutaneous fat. An increase of CD4+ROR*γ*t++ cells was found in liver and abdominal fat of mice fed high fat diet (HFD), in comparison with mice fed normal diet (ND). A Treg increase was found in the subcutaneous adipose tissue. The error bars represent ±1 standard error of the mean (SEM). ^#^Statistically significant difference between mice fed HFD and ND (*p* < 0.05). (b) Fold change of leptin gene expression in liver and abdominal (AAT) and subcutaneous (SAT) adipose tissue. An increase of leptin mRNA was observed in SAT and AAT of mice fed a HFD versus mice fed a ND, with a higher increase in the SAT. Leptin gene expression was not detectable in the liver.

Tregs were significantly increased in the SAT of mice fed HFD* versus* mice fed ND (*p* = 0.02), while no difference was found in AAT or liver (Figure 2(a)). Moreover, an increase of the percentage of the highly polarized Th17 (CD4+ROR*γ*t++) was detected in the liver (*p* = 0.02) and the AAT (*p* = 0.01) of mice fed HFD, in comparison with mice fed ND (Figure 2(a)).

### 3.3. RT-PCR

RT-PCR showed a positive cytokine response to HFD: both IL10 and IL17A were expressed in liver (ΔCt values, resp., 15.5 (15.4–16.5) and 21.7 (21.2–22.3)) and both the AAT (resp., 10.8 (5.1–10.9) and 8 (6.9–8.2)) and the SAT (resp., 11.3 (8–11.7) and 6.4 (5.5–6.9)) of mice fed HFD, while they were nondetectable in mice fed ND. Moreover leptin gene expression was increased in the adipose tissue of mice fed HFD compared to mice fed ND. This increase was more pronounced in the SAT district: in the abdominal district leptin increased 2-fold versus 986-fold in the SAT (Figure 2(b)).

### 3.4. Correlations

In addition the significant correlations (Table 3) between the T-cell modifications and metabolic parameters and histology were investigated. Table 3 shows the significant correlations with their respective correlation coefficient. The AAT-derived CD4+ROR*γ*t++ cells positively correlated with the weight gain and with liver histology: a positive correlation was detected with the NAS as well as with its subscores steatosis, ballooning, and lobular inflammation. The liver-derived CD4+ROR*γ*t++ cells positively correlated with the grade of lobular inflammation. Moreover the SAT-derived Tregs positively correlated with the weight gain, the glucose levels, and liver histology (NAS, ballooning, and inflammation) (Table 3).

## 4. Discussion

There is a growing interest in the role of the immune system as a key contributor to the pathogenesis of NASH and the metabolic syndrome [1]. Given the discordant data on the role of Tregs and Th17 in the development of NASH, we performed a study to further elucidate the differential role of these subsets of cells at the different anatomical sites involved in the pathogenesis of NASH and we analyzed the correlation between the tissue specific variations of these cells and the diet-induced metabolic disturbances.

We used a 36-week HFD mouse model to induce NASH. The histologic features fulfilled the histologic criteria for NASH (defined as the simultaneous presence of steatosis, inflammation, and ballooning). There was also a grade 1 fibrosis in some of them [23]. Moreover the mice fed a HFD were obese and showed an impaired glucose tolerance and a leptin upregulation. Our model hence mimics all features of human NASH [27, 28].

**Figure 3:**
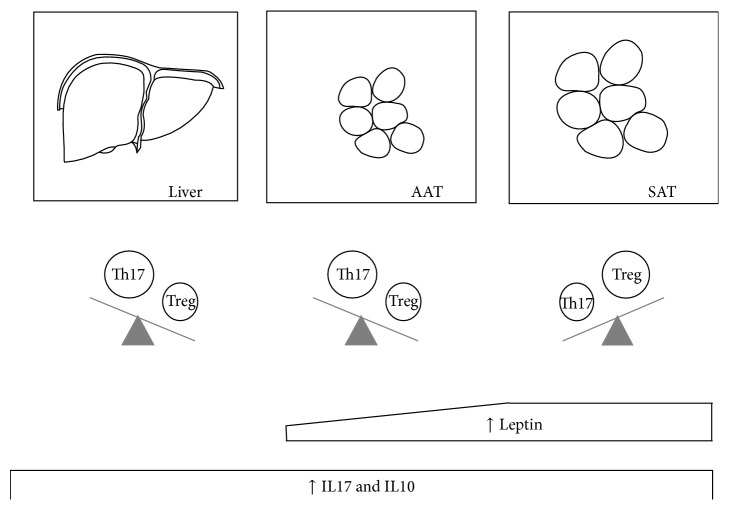
Immune cells, cytokines, and adipokines modifications after high fat diet (HFD). Th17 were significantly increased in liver and abdominal adipose tissue (AAT) of mice fed a HFD while Tregs were significantly increased in the subcutaneous adipose tissue (SAT). After HFD there was an increase in the leptin gene expression in the adipose tissue, more pronounced in the SAT. Moreover there was a positive cytokine response to HFD in liver and adipose tissue (both AAT and SAT).

When considering the different organs investigated (Figure 3), the liver showed an increase of CD4+ROR*γ*t++ cells in mice fed a HFD in comparison with mice fed a ND, while there was no difference in Tregs. Thus, the balance between Th17 and Tregs in the liver was shifted towards Th17. Importantly, the increase in CD4+ROR*γ*t++ cells showed a correlation with liver inflammation (which is an important determinant of liver damage in NASH), but not with metabolic disturbances.

In the AAT, similar to the liver, we observed an increase of the CD4+ROR*γ*t++ in the mice fed a HFD, while Tregs did not show statistically significant variations. Also in this organ HFD shifted the balance between Th17 and Th10 towards the Th17. In line with the active role of the AAT in the onset of NASH and of the metabolic syndrome, the AAT CD4+ROR*γ*t++ correlated with liver histology as well as with some features of metabolic impairment, including weight gain.

In the SAT the Tregs played a predominant role as we observed no statistically significant variations of the CD4+ROR*γ*t++ while the Tregs were significantly increased in the mice fed a HFD. This increase correlated with liver histology impairment and with metabolic alterations, namely, glucose impairment and weight gain.

As far as the Th17 are concerned, our results are in agreement with Tang et al., who demonstrated an increased number of hepatic Th17 and an increased hepatic gene expression in a HFD mouse model, as well as an attenuation of the LPS-induced liver injury after neutralization of IL17 [3]. Others, however, found a decrease of Th17 in the AAT of mice fed HFD [6]. Moreover, in an IL17-deficient mouse model IL17 appeared to act as a negative regulator of adipogenesis and glucose metabolism and to delay the development of diet-induced obesity [5]. This discrepancy could be explained by the different animal models used. In our model, which was able to represent the main characteristics of human NASH, not only in terms of liver histology but also in terms of metabolic impairment, the Th17 were increased in the liver and in the AAT which are key organs in the onset of NASH and positively correlated with liver histology and obesity. This observation, although it cannot mechanistically explain the role of the Th17 in NASH, suggests that Th17 could be key players in NASH and the associated metabolic disease.

Regarding the Tregs, previous preclinical studies reported that AAT is a preferential site of accumulation of Tregs in mice fed a ND and that they decrease after HFD [10, 29]. Others showed an increase of Tregs in the SAT in obesity [29, 30]. In agreement with the latter, our data confirm an increase of the Tregs specifically in the SAT in histologically proven NASH accompanied by metabolic alterations. These data suggest that also the SAT plays an active role in liver disease-related and metabolic-related inflammation. It could also be speculated that in this HFD model the Treg increase in the SAT is an attempt to restore the proinflammatory prone Th17/Treg balance. Further investigations, however, are needed to validate this hypothesis.

Finally we found a leptin upregulation in mice fed a HFD, mainly in the SAT, which, through its systemic effect, may have contributed to the stimulation of immune cells, and particularly the Th17, at distance. This upregulation of the leptin gene expression is in agreement with previous findings [31]. It is known that leptin is able to stimulate the Th17 pathway: in experimental conditions of leptin deficiency, lower levels of IL17 and an impairment of the Th17 pathway are observed while the administration of increasing doses of leptin is able to restore and increase the Th17 pathway [16]. This process could explain, at least in part, the increase of the CD4+ROR*γ*t++ cells in the AAT and the liver as a consequence of increased levels of circulating leptin, mainly originating from the SAT.

In addition leptin has shown proinflammatory, profibrogenic, and prodiabetogenic effects [32]. This is in line with our observation of a significant correlation between leptin and the ALT levels and hepatocellular ballooning, highlighting the link between leptin disturbances and hepatocellular damage.

In line with the ability of leptin to stimulate the inflammatory immune response [33], we found an upregulation of mRNA of IL17A in the liver and adipose tissue of mice fed HFD. These results nicely fit with the aforementioned observed increase of CD4+ROR*γ*t++ in the liver and the abdominal adipose tissue.

In contrast to what could be expected, however, this increase in the proinflammatory IL17A was not counterbalanced by a decrease of the IL10 mRNA in the same sites. Moreover we observed a positive correlation between adipose tissue-derived IL10 and hepatic inflammation at histology. IL10 is an anti-inflammatory cytokine. Its role, however, in diet-induced steatohepatitis and insulin resistance is controversial [34]. Although IL10 has been reported to be protective against diet-induced insulin resistance [35], it has also been reported that IL10 does not improve hepatic or systemic insulin sensitivity in high fat feeding and therefore it does not protect against insulin resistance [36].

In contrast to the flow-cytometric data, the gene expression analysis showed a cytokine upregulation in the liver and in both the AAT and SAT. This discrepancy can be explained by the contribution to cytokine secretion by other cell types, which cannot be discriminated by this method. IL10 increase could be explained, at least in part, due to a tissue-recruitment of M2 macrophages in response to HFD, as previously described [37]. IL17A on the other hand can be produced by cell types other than Th17, such as NKT and *γδ* T lymphocytes [38]. Given the active role of these cells in the onset of NASH and the correlated metabolic disease [39, 40], it could be speculated that also these cells constitute a source of IL17.

In conclusion our results show a stimulation of both the Th17 and the Treg axis in NASH, also in correlation with its metabolic complications. These pathways seem to act differently at different sites, the Th17 axis being upregulated at the level of the AAT and the liver, and the Treg axis being upregulated in the SAT. It has recently been shown that SAT expresses genes that are implicated in inflammation that correlate with liver damage and is therefore also potentially implicated in NASH pathogenesis [41]. The Treg upregulation in SAT observed in our study could hence be as a tentative to increase the anti-inflammatory mechanisms in response to inflammation and, therefore, it could represent a compensatory mechanism to counterbalance the Th17 enhancement. Furthermore, obesity-related overproduction of leptin in SAT could be one of the drivers of the upregulation of the Th17 pathway, further highlighting the potential contribution of the SAT, besides that of the AAT. As this is, however, a cross-sectional study, it is not possible to draw clear cause-effect conclusions. Further functional studies are needed to clarify these aspects.

## Figures and Tables

**Table 1:** Main characteristics of the model.

	ND (*n* = 7)	HFD (*n* = 8)	*p*
Δ weight (g)	5.6 (3–9)	30.6 (12–42)	0.001^*∗*^
Fasting glucose (mg/dL)	101 (85–148)	166 (77–291)	0.02^*∗*^
ALT (UI/L)	30 (20–33)	223 (179–250)	0.01^*∗*^
NAS	0	4.5 (1–6)	0.000004^*∗*^
Steatosis (*n*)			
0	7	1	
1	0	4	
2	0	3	0.000451^*∗*^
Lobular inflammation (*n*)			
0	7	0	
1	0	6	
2	0	2	
3	0	0	0.00002^*∗*^
Ballooning (*n*)			
0	7	1	
1	0	0	
2	0	7	0.000008^*∗*^
Fibrosis (*n*)			
0	7	5	
1	0	3	
2	0	0	0.08

Validation of the model: Δ weight (weight at sacrifice (g) − weight baseline (g)), fasting glucose levels (mg/dL) and histological features (NASH Activity Score (NAS) calculated as the sum of the subscores for steatosis, lobular inflammation, and ballooning) (*n*: number of mice per score). Data are expressed as median and range or as number. ALT: alanine aminotransferase; ND: normal diet; HFD: high fat diet; *p*: *p*-value of the comparison between ND and HFD (Mann-Whitney *U* test and Student's *t*-test, as appropriate). ^*∗*^Statistically significant.

**Table 2:** Flow cytometry analysis.

	Blood %		Liver %		Abdominal fat %		Subcutaneous fat %	
	(median (range))		(median (range))		(median (range))		(median (range))	
	ND	HFD	ND	HFD	ND	HFD	ND	HFD
CD45+	81 (42.3–78.4)	86.1 (69.2–96.3)	66.8 (54.2–78.4)	60 (48.6–71)	35.6 (26.8–73.6)	30.3 (19.8–78.6)	73.9 (32.5–95.61)	74.2 (45.8–46.62)
CD45+CD3+	14 (8.4–11.3)	9.5 (4.9–17.1)	13.6 (7.9–32.4)	18.8 (11.7–46.9)	14.8 (6–61.5)	11.7 (5.4–27.6)	33.1 (13.4–51.6)	24.7 (11.9–28.3)
CD45+CD3+CD4+	42.6 (14.6–39.7)	39.7 (30.1–52.5)	33 (27.4–35.6)	29.1 (9.2–41.4)	45.9 (40.2–56.2)	29.4 (14.7–39.8)^*∗*^	37.4 (28.1–41.6)	41.5 (31.1–46.6)
CD45+CD3+CD4+FOXP3+	9.6 (6.3–10.9)	10 (7.6–15.3)	5.4 (3.3–32.6)	8.5 (4.6–45)	22.8 (13.5–45.6)	16 (10.7–20.1)^*∗*^	8.2 (0.8–13.2)	11.7 (7.6–18.2)
Tregs (CD4+CD25+FOXP3+)	3.5 (1.7–5.5)	3 (2–4.4)	0.14 (0.06–0.6)	0.2 (0.6–3.3)	1.4 (0.6–6.7)	3 (0.6–4.3)	0.6 (0.3–1.3)	1.3 (0.8–3.5)^*∗*^
CD4+ROR*γ*t++	1.7 (0.5–6.7)	2.2 (0.7–5)	2.4 (0.9–5.1)	9.7 (1.5–24.6)^*∗*^	2.3 (0.7–5.8)	8.7 (2.7–15.4)^*∗*^	3.6 (1–18.5)	4.5 (2–10.2)

Flow cytometric analysis of blood, liver, abdominal fat, and subcutaneous fat in mice fed high fat diet (HFD) and normal diet (ND). Cells are represented as % [(median (range)] of the gated population: CD45+CD3+ were gated on CD45+, CD45+CD3+CD4+ were gated on CD45+CD3+, CD45+CD3+CD4+FOXP3+ were gated on the CD45+CD3+CD4+, Tregs (CD45+CD3+CD4+CD25+FOXP3+) were gated on the CD45+CD3+CD4+, and CD45+CD3+CD4+ROR*γ*t++ were gated on the CD45+CD3+CD4+. ^*∗*^Statistically significant difference between mice fed HFD and ND; *p* < 0.05.

**Table 3:** Significant correlations between immune cells and metabolic and histological parameters.

CD4+ROR*γ*t++ abdominal fat and Δweight	*r* = 0.67; *p* = 0.006
CD4+ROR*γ*t++ abdominal fat and NAS	*r* = 0.64; *p* = 0.01
CD4+ROR*γ*t++ abdominal fat and steatosis	*r* = 0.54; *p* = 0.03
CD4+ROR*γ*t++ abdominal fat and ballooning	*r* = 0.55; *p* = 0.03
CD4+ROR*γ*t++ abdominal fat and inflammation	*r* = 0.67; *p* = 0.006
CD4+ROR*γ*t++ liver and inflammation	*r* = 0.64; *p* = 0.01
Treg subcutaneous fat and Δweight	*r* = 0.60; *p* = 0.01
Tregs subcutaneous fat and fasting glucose	*r* = 0.59; *p* = 0.01
Treg subcutaneous fat and ballooning	*r* = 0.57; *p* = 0.02
Treg subcutaneous fat and inflammation	*r* = 0.55; *p* = 0.03
Treg subcutaneous fat and NAS	*r* = 0.52; *p* = 0.04
ΔCT IL10 abdominal fat and inflammation	*r* = −0.92; *p* = 0.008
ΔCT leptin subcutaneous fat and ballooning	*r* = −0.79; *p* = 0.03
ΔCT leptin abdominal fat and ALT	*r* = −0.90; *p* = 0.03

Significant correlations with the respective correlation coefficient (Spearman's rank correlation coefficient, *p* < 0.05; *r* > 0.5). ΔCt: Ct value normalized to the housekeeping gene; NAS: NASH activity score, ALT: alanine aminotransferase.
